# The Sick, the Churches, and the Hospitals

**Published:** 1887-11-12

**Authors:** 


### THE SICK, THE CHURCHES, AND THE HOSPITALS.

[Correspondence is invited.]

The paper on this subject by Sir Andrew Clark, Bart., M.D., published on page 91 of last week's Hospital has excited much genuine interest in hospital circles, as well as amongst the clergy and ministers of religion throughout the metropolis. The Bishop of London, the Right Hon. the Earl of Northbrook, and Lord Sandhurst have already notified their intention to be present at the conference in the early part of 1888, which there is every reason to hope will result in great benefit to many people. It is calculated that in practice the scheme will relieve the clergy and ministers of religion, and their assistants from much present anxiety and worry, whilst it will prove of great advantage to the hospitals as well as to the sick poor. A copy of the scheme and forms of assent have been posted to the clergy of all denominations throughout the metropolis and home counties and to rural deans throughout the country. Anyone who has not received a copy can procure one on application to the *Secretary, The Hospitals Association*, Norfolk House, Norfolk Street, W.C.

### The Conference in 1888.

It is gratifying to find that the clergy and ministers of all denominations are expressing their willingness to attend the conference, with one or two members of their congregations, to be held in January or February next, under the presidency of Sir Andrew Clark, Bart., M.D. The object of the conference will be to formulate proposals to facilitate the carrying out of Sir Andrew's scheme, published in The Hospital for November the 5th. Not only the leaders of religious thought and opinion in the metropolis, but also the clergy generally, as well as eminent laymen and representative hospital managers, are notifying their intention of attending the conference and we propose to publish a list of names on an early date Any clergyman or minister of religion, any hospital manager or governor, who may be desirous of taking part in the conference, is requested to send his name (and in the case of a clergyman that of his church also) to the *Secretary of The Hospitals Association*, Norfolk House, Norfolk Street, W.C., without delay, so that it may be included in the first list published.

